# A Systemic Lupus Erythematosus Patient with Isolated Neutropenia and Diminished Expression of CD55 and CD59 Similar to Paroxysmal Nocturnal Hemoglobinuria

**DOI:** 10.4274/tjh.2013.0318

**Published:** 2014-09-05

**Authors:** Abdülkerim Yıldız, Merih Kızıl Çakar, Elif Suyanı, Gülsan Türköz Sucak

**Affiliations:** 1 Gazi University Faculty of Medicine, Department of Hematology, Ankara, Turkey

**Keywords:** SLE, CD55, CD59, PNH

## TO THE EDITOR

The association of systemic lupus erythematosus (SLE) and paroxysmal nocturnal hemoglobinuria (PNH) has been rarely reported [1,2]. However, diminished expression of CD55 and CD59 on red blood cells and lymphocytes has been demonstrated in SLE patients in the absence of PNH [3,4,5,6].

A 30-year-old female with a history of SLE was diagnosed with pulmonary artery embolism after being admitted to the hospital with sudden-onset dyspnea. She was treated with heparin, which was followed by warfarin. She also had a previous history of deep venous thrombosis of the left lower extremity while on oral contraceptives 2 years prior to the diagnosis of SLE. After the diagnosis of SLE, steroids and hydroxychloroquine were begun, which she stopped taking on her own. Blood cell counts revealed neutropenia with a white blood cell count of 2.32x109/L, neutrophil count of 0.94x109/L, lymphocyte count of 0.93x109/L, hemoglobin of 137 g/L, and platelet count of 254x109/L. Serum biochemistries were within normal limits. Immunological tests revealed positive antinuclear antibodies (3 positive, thin granular, spotted) and anti-dsDNA at a level of 587 IU/mL. Tests for antiphospholipid syndrome including lupus anticoagulants, β2-glycoprotein I antibodies, and anticardiolipin and antiphospholipid antibodies were all negative. Genetic mutations for factor V Leiden and the prothrombin gene were negative. Proteins C and S and antithrombin III antigens were all within the reference range. Flow cytometry analysis showed diminished expression of CD59 and CD55 in the neutrophils and erythrocytes in the absence of a discrete clone (Figures 1a and 1b). The expressions of CD59 and CD55 on lymphocytes were normal. Informed consent was obtained.

PNH is characterized by the deficiency of glycosyl phosphatidylinositol-anchored proteins CD55 and CD59, leading to increased susceptibility to complement-mediated lysis of erythrocytes, leukocytes, and platelets. Diminished expression of CD55 and/or CD59 was previously reported in SLE patients with lymphopenia [4,5] and hemolytic anemia [3,6]. Antibodies against lymphocytes are claimed to cause lymphopenia via mechanisms including antibody-dependent cellular cytotoxicity, opsonization, surface receptor blockage, or apoptosis [5]. However, diminished expression of CD55 and/or CD59 leading to enhanced susceptibility to complement-mediated lysis might also have caused lymphopenia. Our patient had neutropenia with a normal lymphocyte count and did not seem to have PNH as she had no discrete PNH clone, despite diminished expression of CD59 on erythrocytes and CD55-CD59 expression on neutrophils. We think that the diminished expression of complement regulatory proteins in the presented patient might be secondary to SLE-derived autoantibodies, which might lead to complement-mediated cell (neutrophil) lysis and might have contributed to the development of neutropenia. Our patient also had concomitant pulmonary embolism. To the best of our knowledge, diminished expression of CD55 and/or CD59 was not reported before in SLE patients with isolated neutropenia in the absence of PNH.

## CONFLICT OF INTEREST STATEMENT

The authors of this paper have no conflicts of interest, including specific financial interests, relationships, and/ or affiliations relevant to the subject matter or materials included.

## Figures and Tables

**Figure 1a:**
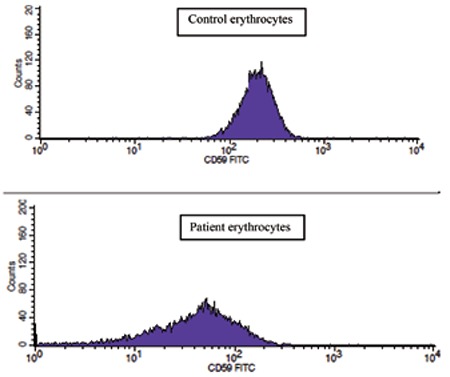
Diminished expression of CD59 on patient erythrocytes.

**Figure 1b:**
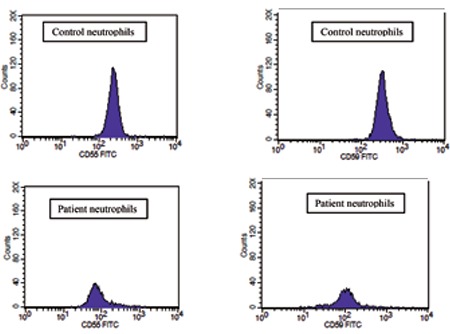
Diminished expression of CD55 and CD59 on patient neutrophils.
